# Neuroprotective Role of Nanoencapsulated Quercetin in Combating Ischemia-Reperfusion Induced Neuronal Damage in Young and Aged Rats

**DOI:** 10.1371/journal.pone.0057735

**Published:** 2013-04-19

**Authors:** Aparajita Ghosh, Sibani Sarkar, Ardhendu K. Mandal, Nirmalendu Das

**Affiliations:** 1 Biomembrane Division, Indian Institute of Chemical Biology, Kolkata, West Bengal, India; 2 Division of Molecular Medicine, Bose Institute, Kolkata, West Bengal, India; Universidad de Castilla-La Mancha, Spain

## Abstract

Cerebral stroke is the leading cause of death and permanent disability among elderly people. In both humans and animals, cerebral ischemia damages the nerve cells in vulnerable regions of the brain, viz., hippocampus, cerebral cortex, cerebellum, and hypothalamus. The present study was conducted to evaluate the therapeutic efficacy of nanoencapsulated quercetin (QC) in combating ischemia-reperfusion-induced neuronal damage in young and aged Swiss Albino rats. Cerebral ischemia was induced by occlusion of the common carotid arteries of both young and aged rats followed by reperfusion. Nanoencapsulated quercetin (2.7 mg/kg b wt) was administered to both groups of animals via oral gavage two hours prior to ischemic insults as well as post-operation till day 3. Cerebral ischemia and 30 min consecutive reperfusion caused a substantial increase in lipid peroxidation, decreased antioxidant enzyme activities and tissue osmolality in different brain regions of both groups of animals. It also decreased mitochondrial membrane microviscosity and increased reactive oxygen species (ROS) generation in different brain regions of young and aged rats. Among the brain regions studied, the hippocampus appeared to be the worst affected region showing increased upregulation of iNOS and caspase-3 activity with decreased neuronal count in the CA1 and CA3 subfields of both young and aged rats. Furthermore, three days of continuous reperfusion after ischemia caused massive damage to neuronal cells. However, it was observed that oral treatment of nanoencapsulated quercetin (2.7 mg/kg b wt) resulted in downregulation of iNOS and caspase-3 activities and improved neuronal count in the hippocampal subfields even 3 days after reperfusion. Moreover, the nanoformulation imparted a significant level of protection in the antioxidant status in different brain regions, thus contributing to a better understanding of the given pathophysiological processes causing ischemic neuronal damage.

## Introduction

Cerebral ischemic diseases are the third most common cause of death and one of the leading causes of senile dementia in the world. The reduction in glucose and oxygen transport to the brain during cerebral ischemic conditions causes bioenergetic failure which leads to oxidative stress, inflammation, blood-brain-barrier (BBB) dysfunction, and eventually leads to cell death [1]. Reactive oxygen and nitrogen species mediated oxidative insults during cerebral ischemia and reperfusion result in damage in vulnerable regions of the brain. Nitric oxide (NO) is an important regulatory molecule for the host defence that plays a vital role in the nervous system. Studies have suggested that large amounts of NO produced by inducible nitric oxide synthase (iNOS) are toxic to the injured brain and contribute to the late stages of cerebral ischemia [2], [3]. Oxidative injury worsens when blood flow is refurbished during reperfusion [4], [5].

Some of the brain regions viz. cerebrum, cerebellum, hypothalamus and hippocampus are more vulnerable to ischemia than others. Severe damage to the hippocampus results in difficulties in forming new memories (ante retrograde amnesia), and often affects memories formed before the damage (retrograde amnesia) [5], [6]. Distinct populations of hippocampal neurons demonstrate differential vulnerability to ischemia [6].

It is necessary to introduce exogenous antioxidants as drugs as well as free radical scavengers to counter cerebral ischemia and reperfusion induced oxidative attack, especially in aged individuals. At present, there are hardly any efficient neuroprotective agents that may be used for treating ischemic stroke, especially in the elderly. Quercetin (QC) is an important bioflavonoid polyphenolic antioxidant and it can be found in fairly large amounts in fruits, vegetable oils, red wine, and tea [7], [8] and is known as a strong free radical scavenger [9]. However, several studies have shown that when applied exogenously, antioxidants are unable to attenuate cerebral ischemic injuries [10], [11], [12], [13]. Although Quercetin is a well known bioflavonoid for its multiple medicinal benefits, it cannot cross the BBB due to its water insolubility and low oral bioavailability, a major stumbling block in CNS therapeutics. Therefore, it becomes necessary to develop a system which could provide an elevated pool of such bioflavonoid antioxidants in the brain for complete protection of neuronal cells against oxidative attack.

Long circulating polymeric nanoparticles made of polylactide-co-glycolide (PLGA) are well accepted as effective drug carriers because of various technological advantages viz., long shelf life, high carrier capacity and feasibility of various routes of administration including oral route [14]. Moreover, nanoparticles have a good safety profile, can cross the BBB and provide drug-sustained release [15]. Polymeric nanoparticles in CNS targeted drug delivery provide better penetration of therapeutic agents, and a reduced risk in comparison to existing therapies [16]. By using PLGA nanoparticulated drug delivery vehicles it is possible to deliver the drug to the targeted tissue across the BBB, release the drug at a controlled rate, and avoid degradation processes.

The aim of our present study is to ascertain whether oral treatment with PLGA nanoparticulated quercetin exert any neuroprotective effect against cerebral-ischemia reperfusion evoked oxidative damage in the neuronal cells in different brain regions, especially the hippocampus, of young and aged rats.

## Materials and Methods

### Chemicals

Polylactide-co-glycolide (PLGA;Resomer RG 85∶50H) didodecyldimethylammonium bromide (DMAB) and quercetin were purchased from Sigma – Aldrich (St. Louis, MO, USA). Ethyl acetate (AR grade) was purchased from Rankem Fine Chemicals (New Delhi, India). Chloroform and methanol were purchased from E. Merck. All other reagents were of analytical grade.

### Preparation of nanoparticle encapsulated quercetin

A modified emulsion – diffusion – evaporation method [17] was used to prepare quercetin nanocapsules. In brief, 36 mg of PLGA was dissolved in 2.5 ml of ethyl acetate at room temperature. Quercetin was dissolved in ethyl acetate. The organic solution of PLGA and drug in ethyl acetate was then emulsified with 5 ml of an aqueous phase containing DMAB. The resulting organic/water emulsion was stirred at room temperature for 3 h before being homogenized at 15,000 rpm for 5 min with a high-speed homogenizer (Polytron PT4000; Polytron Kinematica, Lucerne, Switzerland). The organic solvent was removed by constant stirring on a water bath set at 40°C. The suspension was ultracentrifuged at 105,000 g in a Sorval RC 5B Plus using the Sorval T-865 rotor for 1 h. The pellet of nanocapsules was washed with phosphate-buffered saline (PBS) twice and resuspended in 2 ml PBS. The nanosuspension was then lyophilized and stored at −20°C for future use.

### Characterization of nanoparticles encapsulated quercetin using Transmission Electron Microscopy (TEM) and Atomic Force Microscopy (AFM)

The Transmission Electron Microscopy observations were performed with TECNAI G2 BIOTWIN system at a magnification of 9.9X. Initially 10 μl of the nanoencapsulated quercetin suspension was retrieved and placed upon 300 mesh carbon coated copper grids. The grids containing the samples were dried extensively under lamp. The grids were then stained with 2% Phosphotungstic acid solution followed by vigorous washing with thin flow of MilliQ water. The stained grids were then dried again, inserted in the sample receiver and allowed to wait until the machine created a vacuum before obtaining images. The images of the samples were transferred to the computer screen of the instrument and the particle diameter was measured using the TEM Software program at different magnifications [18]. The atomic force microscopy (AFM) observations were performed with a picoview 1.10.4 version AFM system (Agilent Technologies, USA). All the images were obtained in the Aquastic mode using cantilevers having a resonance frequency of 146–236 kHz, tip height 10–15 μm, and tip length 225 μm. Mica was chosen as a solid substrate and used immediately after cleavage in a clean atmosphere. During the characterization experiment, the probe and cantilever were immersed completely in the water solution. The nanoparticulated quercetin suspensions on mica were dried in air (65% humidity) for 30 min. Images were analyzed with the help of Pico Image Software from Agilent Technologies, USA [19].

### Study design in animal model

Male Sprague Dawley rats of two age groups, young (2 months) and aged (20 months) weighing 150–170 g and 415–440 g respectively, were used. The animals were kept in a temperature and humidity controlled housing with 12 hrs light and dark cycles. They were acclimatized for 3–5 days to the new environment before use and allowed free access to food and water. Rats from each category (2 months old and 20 months old) were subdivided into nine groups, each group consisting of 6 animals. Two groups of animals were used for saline treatment (normal group and sham operated group). Three experimental groups of rats (both young and aged) were respectively treated with free quercetin (2.7 mg/kg b wt), empty PLGA nanoparticles (2.7 mg/kg b wt) and PLGA nanoparticulated quercetin (2.7 mg/kg b wt) via oral gavage, two hours prior to ischemic induction. Rest of the four experimental groups of rats (both young and aged) were treated with free quercetin (2.7 mg/kg b wt) and nanoparticulated quercetin (2.7 mg/kg b wt) till day 1 and day 3 post-operation respectively via oral gavage. All the rats used in this study received proper care in compliance with the Institutional Animal Ethics Committee (IAEC), Indian Institute of Chemical Biology, India, Registration No. 147/99/CPC SEA and the Committee specifically approved this study.

### Induction of cerebral ischemia-reperfusion

Rats from both young and aged groups (excluding normal groups) were anesthetized by a single intraperitoneal injection of Urethan (35 mg/kg) and were exposed to ischemia by bilateral clamping of the common carotid arteries. After 30 min. of ischemic insult, animals were further subdivided into three groups (both young and aged) subjected to reperfusion (by withdrawing clamps) intervals of 30 min, 24 h and 72 h respectively. The animals that underwent reperfusion for 24 h and 72 h were given appropriate postoperative care till day 3. After the respective reperfusion intervals, all the animals were killed by decapitation [20]. Only the hippocampal region of the later groups of rats was isolated for a detail study regarding the effect of nanoencapsulated quercetin on delayed neuronal death due to cerebral ischemia.

The brain tissues of all the other animals were isolated immediately and rinsed with ice-cold saline. Different brain regions viz. hippocampus, hypothalamus, cerebral cortex and cerebellum were isolated. Small portions of all these brain regions were also kept for the analysis of edema development. The brain regions were then homogenized in phosphate buffer saline with Teflon coated homogenizer for different biochemical assays.

### Measurement of ROS level

Intracellular ROS level of different brain regions were measured [21]. Briefly, all the four brain regions were homogenized (10%) in PBS (pH 7.2). The homogenized cells (0.4 mg/ml) were loaded with the cell permeant probe CM-H_2_DCFDA (5-(and-6)- chloromethyl-2′,7′-dichlorodihydro-fluorescein diacetate acetyl ester) (2 μM) for 15 min at 30°C in dark, and fluorescence was monitored. H_2_DCFDA, an uncharged, cell permeable fluorescent probe readily diffuses into cells and gets hydrolyzed by intracellular esterases to yield H_2_DCF, which is trapped inside the cell. Then it is oxidized from the non-fluorescent form to a highly fluorescent compound dichlorofluorescein primarily by hydroxyl radical (•OH), hydrogen peroxide (H_2_O_2_) or other low-molecular-weight peroxides produced in the cells. Thus the fluorescence intensity is proportional to the amount to the ROS produced by the cells. Fluorescence was measured through a spectrofluorometer (LS 3B, Perkin Elmer, USA) by using 499 nm as excitation and 520 nm as emission wavelengths. The data were normalized to normal values, and the normal was expressed as a value of 100%.

### Lipid peroxidation assay in brain homogenate

Lipid peroxidation in different brain regions of experimental rats was determined by measuring the amount of conjugated diene. The brain tissue homogenates were extracted in a chloroform-methanol mixture (2∶1,v:v), and the extracted lipid solvent mixture was evaporated to dryness under nitrogen atmosphere at 25°C and dissolved in cyclohexane. The lipid content in cyclohexane was assayed at 234 nm, and the results were expressed as μmol lipohydroperoxide/mg protein by using a εm of 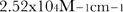 [22]. Proteins were measured according to Lowry et al. (1951).

### Reduced Glutathione (GSH) assay

The intracellular GSH content in different brain regions was assayed at the end of the specified time. Samples of the brain tissues were removed from each animal, rinsed in ice-cold saline and blotted carefully. The samples were placed in a previously weighed ice-cold glass homogenizer containing phosphate buffer (pH 7). The weight of the added sample was determined. After homogenization, an equal volume of 10% metaphosphoric acid was added and mixed by vortexing. The mixture was allowed to stand for 5 min at room temperature. After centrifugation for 5 min, the supernatant was collected carefully without disturbing the precipitate. The GSH contents of the neutralized supernatant were assayed using Ellman's Reagent [5,5′-dithiobis-2- nitrobenzoic acid (DTNB)] according to the method described by Griffith (1980) [24]. A standard reference curve was prepared for each assay.

### Assay of cytosolic enzymes

Remaining portions of the brain regions were homogenized in 0.25 M sucrose with Teflon coated homogenizer and centrifuged at 10,000 g for 15 min. A portion of supernatant was further centrifuged at 1,05,000 g for 60 min. The supernatant from the second centrifugation contains the cytosolic fraction of brain cells [25]. The catalase activity in young and aged rat brain regions was measured spectrophotometrically by calculating the rate of degradation of H_2_O_2_ [26]. Superoxide dismutase (EC1.15.1.1) activity (SOD) in cytosolic fraction of brain homogenate of all the groups of rats (young and aged) was estimated by following the method of Murkland and Murkland [27].

### Quantitation of cerebral edema

The entry of plasma water into swollen brain of young and aged rats has been calculated by the resultant change in cerebral tissue osmolality by the induction of cerebral ischemia – reperfusion. Comparative studies in brain tissue osmolality have been performed among normal, ischemia-reperfused and other groups of ischemia-reperfused rats pre-treated either with free QC (2.7 mg/kg b.w.) or mannosylated liposome entrapped QC by the following the equation [28].


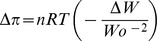


Where ΔII represents the resultant change in tissue osmolality, n denotes the osmols in an initial weight of water W0 (g) of brain tissue; DW, an additional amount of water (g) enters into brain tissue and RT =  {1.83–107(mmHg) cm3/mol} indicates the product of gas constant and absolute temperature.

### Western Blot to detect iNOS

The hippocampal regions were dissected out from both groups of animals, were homogenized with a Dounce homogenizer in lysis buffer containing 100mM Tris (pH 7.4), 150 mM NaCl, 1% Triton X-100, 1% sodium deoxicholate, 0.1% sodium dodecylsulphate (SDS), 5 mM EDTA, 1mM phenyl-methylsulfonyl fluoride, 5 μg/ml protease inhibitor cocktail at 4°C. The homogenates were centrifuged at 15,000× g for 15 min. The supernatants were used to determine the total protein content [23]. Cytosolic fractions thus prepared from hippocampus of experimental rats were mixed with Laemmli's dye, boiled for 5 mins, and subjected to 12.5% SDS-PAGE at 100 volts followed by electroblotting to PVDF membrane for 20 mins. At 15 volts membranes were blocked with 5% non fat milk in phosphate-buffered saline overnight at 4°C. The membranes were subsequently washed with tween 20 in phosphate buffered saline and probed with mouse anti-iNOS (1∶2000, Santa Cruz) antibody for 3 hours. The membranes were again washed in the same manner and incubated with an alkaline phosphatase conjugated antirabbit IgG (1∶30,000). Following the secondary antibody incubation, the membranes were rinsed and bound antibodies were detected using BCIP-NBT salts [29]. For quantification, the pixel intensities of the respective bands of the immunoblots from six different individual rats were calculated and analysed using Image J software (NIH, USA). The intensities of the bands were expressed as relative pixel density (arbitrary units).

### Fluorescence depolarization measurements of the fluidity of mitochondrial membrane

The fluorescence depolarization, associated with the hydrophobic fluorescence probe diphenyl hexatriene (DPH), was used to monitor the changes in the fluidity of the lipid matrix accompanying the gel to liquid crystalline phase transition. The mitochondrial membrane fraction was incubated at 37°C by the addition of DPH dissolved in tetrahydrofuran (DPH: lipid molar ratio, 1∶500). The excitation and emission maxima were 365 and 430 nm, respectively. The fluorescence anisotropy was calculated by using the equation:
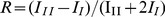
(1)where, I_II_ and I_I_ are the fluorescence intensities parallel and perpendicular to the direction of the excited light. The microviscosity parameters [(*r*_0_/*r*) − 1]^−1^ were calculated in each case using 0.362 as the maximal limiting fluorescence anisotropy for DPH [30].

### Caspase-3-like enzyme activity

Rats were anaesthetized and after they underwent ischemia-reperfusion, they were sacrificed. A predetermined region of injured hippocampus was dissected, weighed and stored at −70°C for batch analysis. The tissue was then homogenized in cell Lysis buffer and the homogenate was centrifuged first at 6000 rpm for 10 min and the supernatant was again subjected to centrifugation at 1,05000 rpm for 1 hr. The supernatant was collected and used as the cytosolic preparation for further analysis of Caspase-3 activity using Caspase-3 Assay kit from Sigma-Aldrich. 100 μg of cytosolic protein was diluted with 50 μl of cell lysis buffer. 50 μl of 2× Reaction buffer containing 10 mM DTT was added to each sample. Each sample was assayed with and without addition of 5 μl of the caspase-3- like tetrapeptide substrate DEVDp- nitroanilide. Samples were incubated at 37°C for 1 hr. Cleavage of the chromophore from the substrate was detected spectrophotometrically at a wavelength of 405 nm [31]. Caspase-3 activity was calculated in μmol of pNA released per min per ml of cell lysate or positive control based on the formula:
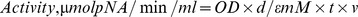
Where: εmM  = 10.5,

v – Volume of sample in ml,

d – Dilution factor,

t – Reaction time in minutes.

### Hippocampal pyramidal neuron density of young and aged rats

The left and right hippocampal regions of both normal and experimental groups of young and aged rats were dissected out after their sacrifice and fixed by immersion in formalin solution. The tissue block was trimmed until it was 1cm thick and was then processed into paraffin wax. One hundred serial 5 μm sections were cut perpendicular to the long axis of the hippocampus. Every tenth section was taken, mounted onto glass slides and stained with cresyl violet [32]. Two continuous fields in hippocampal CA1 and CA3 subfields were selected for each section and the number of nucleolated pyramidal neurons within it counted at 20× magnification with a fitted calibrated eyepiece graticule. These measurements were made ‘blind’ without knowledge of the section's regional origin or history. The sections were then decoded and the mean pyramidal neuron density/mm3 within CA1 and CA3 subfields of both young and aged rat hippocampus was calculated as follows:
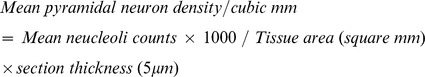



### Statistical analysis

Mean and SEM were calculated for all data. Significant difference between mean were evaluated by analysis of variances. Linear correlations between two data were calculated by means of Pearson's correlation coefficient *r*. A difference was considered significant when p was less than 0.05.

## Results

### Nanocapsule characterization by Transmission Electron Microscopy (TEM) and Atomic Force Microscopy (AFM)

**Figure 1:**
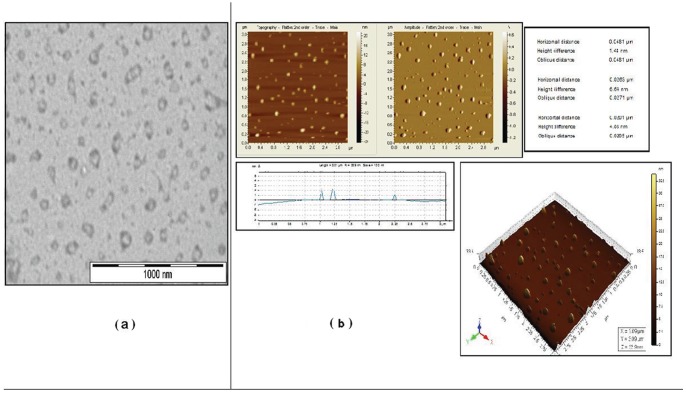
TEM and AFM images of nanocapsules. **a.** TEM pictures of Nanoparticulated quercetin at a magnification of 20,500X demonstrate particles with a spherical morphology and an average size distribution from 10–50 nm. **b.** AFM images of nanocapsules obtained 2 min after deposition on mica support. Amplitude-flattened view of nanoparticlulated quercetin is shown. Horizontal cross section indicates the height of the nanocapsules from the substratum, i.e., the mica sheet.

The nanocapsules obtained were in the size range 20–50 nm. The TEM and AFM images (Fig. 1) revealed that nanocapsules were spherical in shape with a narrow size distribution. Encapsulation of quercetin in nanocapsule was found out to be about 80%. The percentage encapsulation of a drug delivery system determines the amounts of drug and excipient that a dose incorporates. Percentage encapsulation information was used to calculate the amount of nanocapsule suspension to be administered to the animals.

### Effect of nanoencapsulated quercetin on age related conjugated diene generation in different brain regions due to induction of cerebral ischemia and reperfusion

Lipid peroxidation is a major marker for stress induced membrane damage. The mean ± S.E. values of conjugated diene that are generated in different brain regions of young and aged rats by the induction of cerebral ischemia and reperfusion is summarized in Table 1. In young rats, short term ischemia (30 min) followed by consecutive 30 min reperfusion causes a substantial increase in diene level in different brain regions examined, the highest level was found in the hippocampus (5.04±0.040) and the lowest in the cerebellum (3.42±0.104) (Table 1). Cerebral cortex (4.14±0.196) and hypothalamus (3.59±0.060) also experienced significant thrust in diene level. In aged rats also, cerebral ischemia and reperfusion caused a massive increase in the diene level in brain, the highest level again being the hippocampus (6.78±0.237 µmol/mg protein). Pre-treatment of rats with free quercetin (2.7 mg/kg b.wt), two hours prior to induction of ischemia resulted in almost no significant protection against cerebral ischemia and reperfusion – mediated diene generation in brain regions of both young and aged rats. But conjugated diene generation in different brain regions of the ischemic rats was markedly reduced almost to the normal levels, by a single dose oral treatment of nanoencapsulated quercetin (2.7 mg/kg b. wt.). Best results were obtained in the hippocampal region in young (0.91+0.050) and aged (3.43+0.050) animals. In the case of normal aged animals, the diene level was found to be higher in all the brain regions in comparison to that of normal young animals.

**Table 1:** Effect of nanocapsulated quercetin on conjugated diene in different brain regions of ischemia-reperfusion induced young and aged rats.

Experimental Conditions	*Lipohydroperoxide content (μ mol/mg protein)*							
	*Young rats*				*Aged rats*			
	Cerebral cortex	Cere-bellum	Hypo- thalamus	Hippo- campus	Cerebral Cortex	Cere-bellum	Hypo- thalamus	Hippo- campus
***Normal***	1.06± 0.035	0.95±0.051	1.05±0.052	0.91±0.063	3.54±0.122	3.06±0.040	3.03±0.027	3.62±0.077
Cerebral-ischemia reperfused(**A**)	4.14±0.196^*^	3.42±0.104^*^	3.59±0.060^*^	5.04±0.040^*^	5.60±0.037^*^	5.27±0.094^*^	5.27±0.070^*^	6.78±0.237^*^
**(A)+Free Quercetin (QC) treated**	3.74±0.192	3.38±0.034	3.37±0.040	5.28±0.095	5.44±0.040	5.24±0.0.058	5.23±0.057	6.79±0.177
**(A)+Empty Nanoparticles**	3.21±0.154	3.10±0.027	2.99±0.052	5.12±0.072	5.07±0.039	5.05±0.047	5.07±0.051	6.32± 0.112
**(A)+Nanocap-sulated QC Treated**	0.93±0.050^**^	0.92±0.027^**^	0.89±0.037^**^	0.91±0.050^**^	3.25±0.079^**^	3.07±0.057^**^	3.17±0.047^**^	3.43±0.050^**^

Values are mean ± SE of rats. *,P<0.001 significantly different from normal; **,P<0.001 significantly different from ischemia-reperfused rats treated.

### Effect of nanoencapsulated quercetin on cerebral ischemia-reperfusion induced antioxidant defences in young and aged rat brain regions

**Figure 2:**
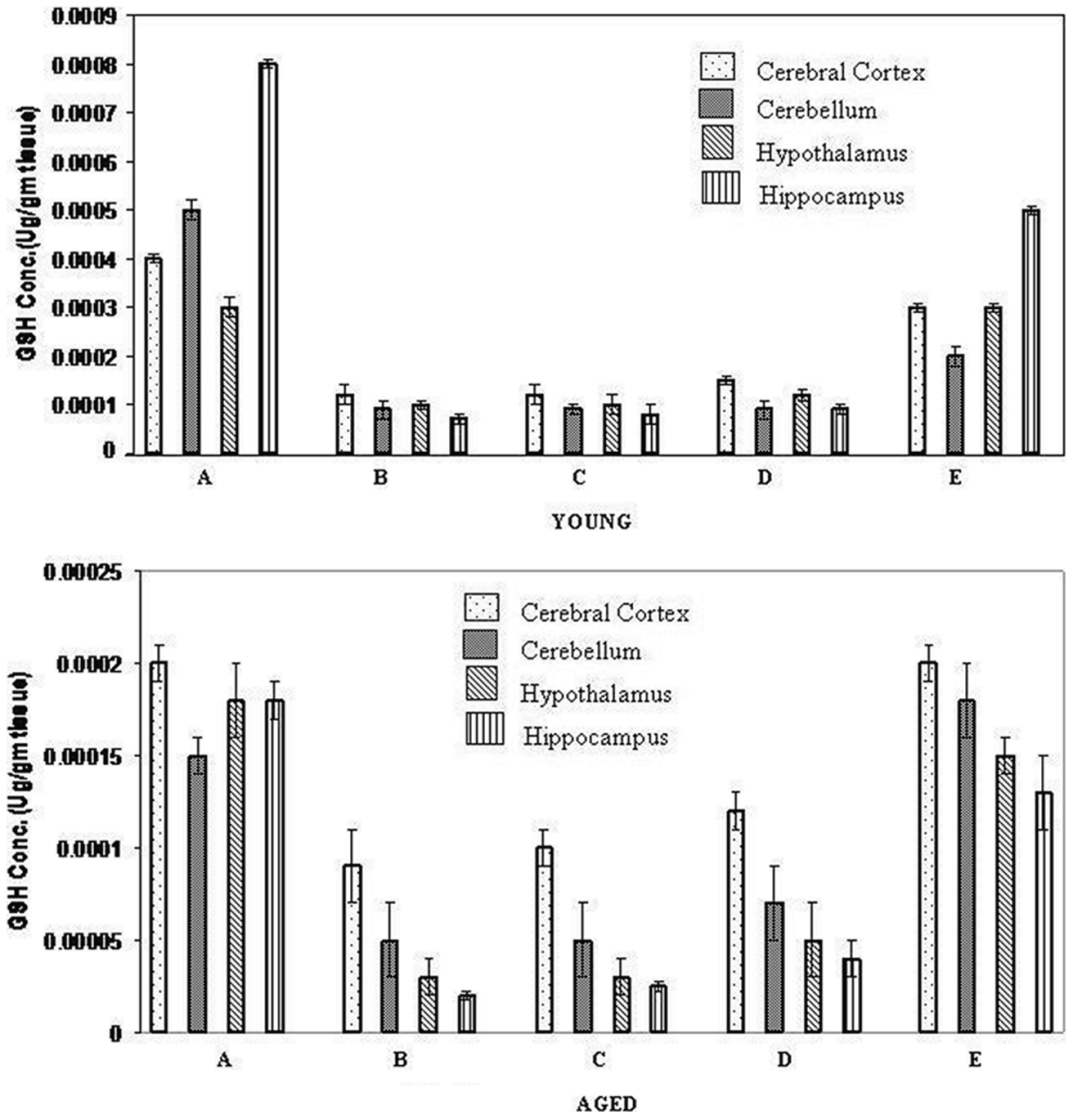
Effect of nanoencapsulated quercetin on GSH concentration in different brain regions of ischemia-reperfusion induced young and aged rats. **A**. Normal, **B**. Ischemia-Reperfusion induced, **C**. **B**+Free QC treated, **D**. **B**+ Empty nanoparticle treated, **E**. **B**+ Nano QC treated. Values are mean ± SE of rats.

Age related alteration in different antioxidant enzymes viz. superoxide dismutase (SOD) and catalase in different brain regions by the induction of cerebral ischemia and reperfusion is shown in Table 2. Both SOD and catalase activities were reduced significantly in all the brain regions, especially the hippocampal region (18.70±0.204 and 0.212±0.003 simultaneously) of normal aged rats when compared to that of normal young rats (66.16±1.024 and 0.418±0.003 simultaneously). Ischemia-reperfusion induced a further reduction both in SOD and catalase activities in the aged variety mostly in the cortex (8.36±0.362) and hippocampal (9.37±0.090) regions when compared to those respective regions (26.83±1.633; 17.48±0.714) of the young groups. Free quercetin treatment prior to ischemic insult resulted in no significant protection of those endogenous antioxidants in brain regions of aged or young rats. But treatment with nanoencapsulated quercetin induced complete protection to these enzymes both in young and aged rat brain against any degradation of these enzymes from ischemic insult. Glutathione, the most important intracellular antioxidant, significantly decreased in the brain regions of both young and aged group of rats upon cerebral-ischemia reperfusion, the most affected region being the hippocampus (Fig. 2). The cellular GSH content was significantly increased and brought towards its normal value in both groups of young and aged rats that received nanoparticulated quercetin (2.7 mg/kg. b. wt.) prior to ischemic insult, whereas free quercetin resulted in no significant effect in the brain regions of both young and aged group of rats subjected to ischemia-reperfusion (Fig. 2).

**Table 2:** Effect of nanocapsulated quercetin on the decreased antioxidant enzyme activities in different brain regions of ischemia-reperfusion induced young and aged rats.

Experimental condition	Superoxide dismutase (Percentile Autoxidaion of Pyrogallol)								Catalase (µmol/mg protein)							
	Young rats				Aged rats				Young rats				Aged rats			
	Cerebralcortex	Cerebellum	Hypothala- mus	Hippocamp- us	Cereb-ral -cortex	Cerebellum	Hypotha- lamus	Hippoca- mpus	Cerebral- cortex	Cereb- ellum	Hypotha- lamus	Hippoca- mpus	Cerebral- cortex	Cereb- ellum	Hypotha- lamus	Hippocamp- us
**Normal**	50.69±3.925	63.94±0.827	50.94±1.497	66.16±1.024	26.05±1.213	23.92±0.706	24.74±0.477	18.7±0.204	0.421±0.004	0.442±0.006	0.441±0.005	0.418±0.003	0.261±0.004	0.270±0.005	0.257±0.004	0.212±0.003
**Cerebral Ischemia reperfused (A)**	26.83±1.633	22.38±1.312	19.37±0.770	17.48±0.714	8.36±0.362	12.15±0.392	14.26±0.425	9.37± 0.090	0.240±0.006	0.239±0.004	0.189±0.006	0.191±0.006	0.163±0.007	0.168±0.004	0.171±0.005	0.153±0.006
**(A)+Free QC treated**	28.17±1.454	21.75±1.427	20.94±0.520	18.76±0.630	11.45±1.281	13.44±0.142	15.15±0.454	11.65±1.340	0.273±0.007	0.250±0.004	0.260±0.004	0.199±0.004	0.179±0.003	0.204±0.004	0.176±0.003	0.153±0.006
**A+Empty Nanoparti- cle**	27.78±1.232	20.14±0.453	19.97±0.512	17.92±1.230	10.98±1.231	12.89±0.137	14.79±0.521	11.21±1.211	0.299±0.008	0.220±0.005	0.297±0.003	0.223±0.003	0.198±0.002	0.219±0.004	0.196±0.003	0.162±0.005
**A+Nanocap-sulated QC**	49.39±3.168	49.75±1.544	50.54±0.912	51.84±1.440	20.21±0.579	22.31±0.375	22.18±1.377	13.19±0.358	0.388±0.004	0.444±0.004	0.381±0.008	0.353±0.006	0.243±0.003	0.249±0.004	0.238±0.005	0.198±0.005

Values are expressed as mean ± S.E of rats.

### Effect of nanoencapsulated quercetin on age related changes in cerebral tissue osmolality caused by induction of ischemia and reperfusion

**Figure 3:**
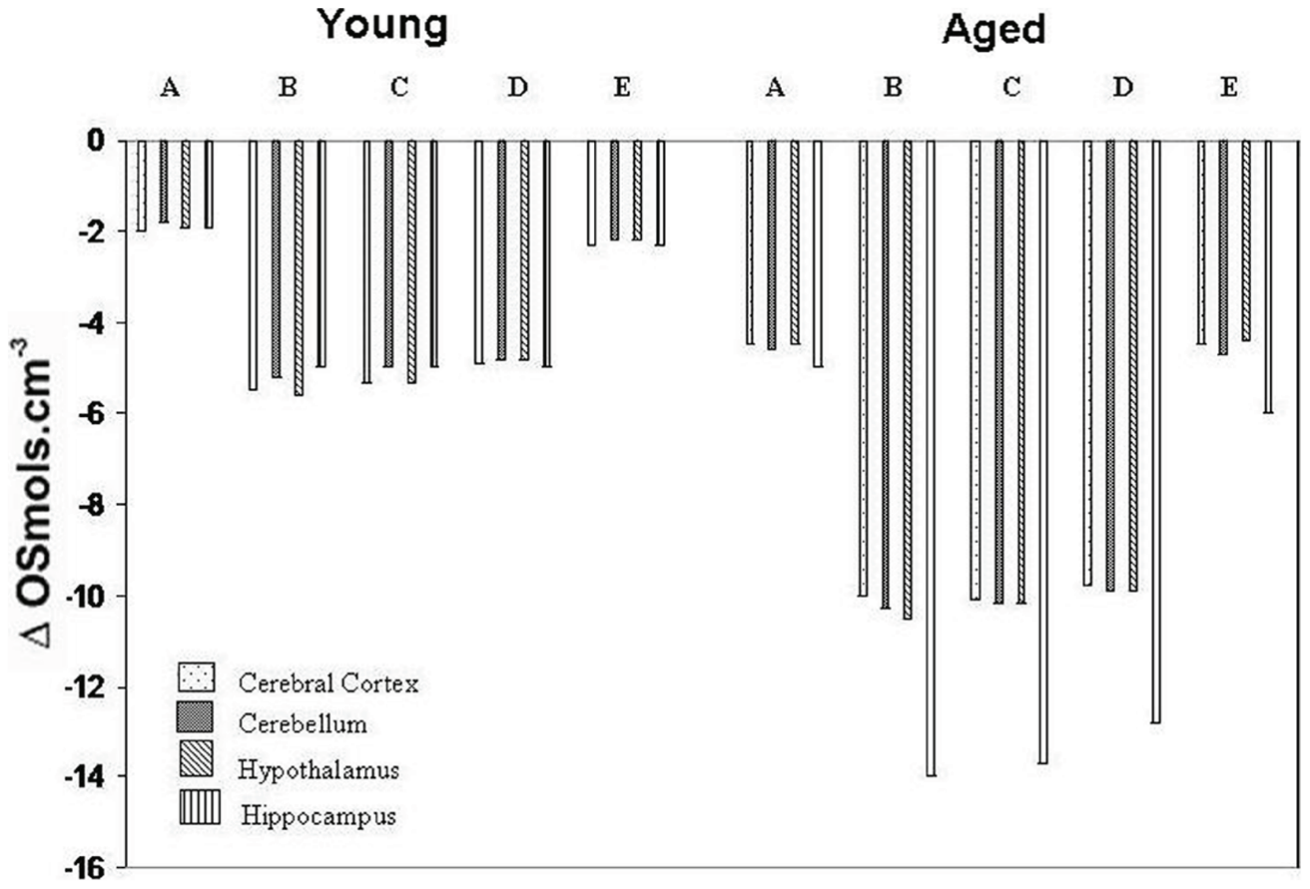
Effect of nanoencapsulated quercetin on cerebral tissue osmolality in different brain regions of ischemia-reperfusion induced young and aged rats. **A**. Normal, **B**. Ischemia-Reperfusion induced, **C**. **B**+ Free QC treated, **D**. **B**+ Empty nanoparticle treated, **E**. **B**+ Nano QC treated Values are mean ± SE of rats.

Ischemic brain edema results from both cell swelling (cytotoxic edema) and increased blood vessel permeability (vasogenic edema). A change in brain tissue osmolality is the index of cerebral edema. Cerebral tissue osmolality is the index of cerebral edema. Cerebral tissue osmolality decreased with development of cerebral edema in different brain regions of both young and aged rats after the induction of ischemia and reperfusion (Fig. 3). Treatment of rats (young and old) with nanoencapsulated quercetin resulted in an increase in brain tissue osmolality whereas free quercetin showed no significant improvement.

### Effect of nanoencapsulated quercetin on ROS generation of ischemia-reperfusion induced different brain regions of young and aged rats

In ischemic brain tissue, ROS generation is accelerated by cytosolic pro-oxidant enzymes and failure to adequately replenish antioxidants. These overproduced ROS cause macromolecular damage and activation of various pathways. In this study, the generated ROS were assessed with the membrane-permeable fluorescent probe 2′,7′-dichlorodihydrofluorescien diacetate (H2DCFDA). Table 3 summarizes mean ± SE values for the oxidized fluorescent product dichlorofluorescein (DCF) fluorescence (marker for ROS generation) of different brain regions of both control and experimental groups of young and aged rats. In the case of normal aged animals, the ROS level was found to be higher in all the brain regions in comparison to that of normal young animals. In young rats, short term ischemia (30 min) followed by consecutive 30 min reperfusion resulted in substantial increase in ROS level in different brain regions examined, the highest level was observed to be in the hippocampus (478.33±0.869% of normal). Cerebral cortex and hypothalamus also experienced significant thrust in ROS level. Pre-treatment of rats with free quercetin (2.7 mg/kg b.wt), two hours prior to ischemic induction resulted in almost no significant protection against cerebral ischemia and reperfusion mediated ROS generation in brain regions both in young and old animals. ROS generation in different brain regions of both groups of animals was markedly reduced almost to the normal levels, by a single oral treatment with nanocapsulated quercetin (2.7 mg/kg b. wt.).

**Table 3:** Effect of nanocapsulated quercetin on ROS generation (DCF Fluorescence) in different brain regions of ischemia-reperfusion induced young and aged rats.

Experimental Conditions	DCF-Fluorescence (% of normal) of different brain regions of young and aged rats							
	Young rats				Aged rats			
	Cerebral Cortex	Cerebe- llum	Hypoth- alamus	Hippo campus	Cerebral cortex	Cere- bellum	Hypothalamus	Hippo campus
**Normal**	101.86±0.844	101.65±0.831	103.81±0.836	102.97±0.841	109.8±0.894	106.6±0.671	107.83±0.618	115.88±0.969
**Cerebral ischemia reperfused (A)**	431.77±0.849^*^	433.5±0.845^*^	445.67±0.837^*^	478.33±0.869	502.7±0.832^*^	504.3±0.551^*^	507.89±0.771^*^	531.72±0.912^*^
**(A)+Free QC treated**	427.54±0.824	429.43±0.681	440.79±0.857	466.44±0.776	501.4±0.885	504.3±0.551	505.92±0.662	514.36±0.763
**(A)+Empty Nanoparticles**	392.54±0.541	397.26±0.621	413.74±0.431	429.37±0.321	443.7±0.667	489.4±0.427	487.57±0.461	477.64±0.459
**(A)+Nano capsulated QC treated**	110.56±0.808**	108.66±0.809**	108.71±0.809**	112.82±0.875^**^	113.3±0.823^**^	111.2±0.859^**^	114.56±0.860^**^	125.92±0.979^**^

Values are mean ± SE of rats. *,P<0.001 significantly different from normal; **, P<0.001 significantly different from ischemia-reperfused rats treated.

### Effect of nanoencapsulated quercetin on mitochondrial membrane microviscosity of ischemia-reperfusion induced different brain regions of young and aged rats

The fluidity of mitochondrial membranes is especially crucial for the production of energy. In the present study, cerebral ischemic insult resulted in reduction in the mitochondrial membrane microviscosity in different brain regions of young and aged rats (Table 4), the worst affected area being the hippocampus. Free quercetin treatment resulted in no significant improvement in mitochondrial membrane microviscosity, whereas nanocapsulated quercetin treatment imparted complete protection to the mitochondrial membrane of all the brain regions in both young and aged rats (Table 4).

**Table 4:** Effect of nanocapsulated quercetin on the mitochondrial membrane microviscosity ([*r*_0_/*r*-1]^−1^) of different brain regions of ischemia-reperfusion induced aged and young rats.

Experimental Conditions	Membrane Microviscosity ([*r*_0_/*r* − 1]^− 1^) of Rat Brain regions							
	Young rats				Aged rats			
	Cerebral Cortex	Cerebe- llum	Hypoth- alamus	Hippo campus	Cerebral Cortex	Cerebellum	Hypoth alamus	Hippocampus
**Normal**	0.571±0.0008	0.569±0.0007	0.575±0.0008	0.573±0.0008	0.542±0.0007	0.539±0.0007	0.547±0.0007	0.526±0.0007
**Cerebral ischemia reperfused (A)**	0.261±0.0007^*^	0.265±0.0008^*^	0.270±0.0008^*^	0.223±0.0007^*^	0.198±0.0009^*^	0.191±0.0009^*^	0.201±0.0008^*^	0.162±0.0009^*^
**(A)+Free QC treated**	0.263±0.0007	0.266±0.0008	0.274±0.0008	0.224±0.0007	0.203±0.0009	0.195±0.0009	0.205±0.0008	0.162±0.0009
**(A)+Empty Nanoparticles**	0.289±0.0006	0.294±0.0005	0.296±0.0007	0.256±0.0008	0.258±0.0004	0.227±0.0006	0.236±0.0005	0.194±0.0007
**(A)+Nano capsulated QC treated**	0.569±0.0008^**^	0.565±0.0007^**^	0.572±0.0008^**^	0.562±0.0007^**^	0.530±0.0007^**^	0.531±0.0007**	0.538±0.0008**	0.514±0.0007**

Values are mean ± SE of rats. *,P<0.001 significantly different from normal; **, P<0.001 significantly different from ischemia-reperfused rats treated.

### Effect of nanoencapsulated quercetin on iNOS protein expression in hippocampal region of young and aged rats

After studying the effects of ischemia and reperfusion in four different vital brain regions of both age groups of animals, we observed that the hippocampus was the most affected amongst all other brain regions. In view of this, we conducted our further studies on hippocampal region of both young and aged rats.

**Figure 4:**
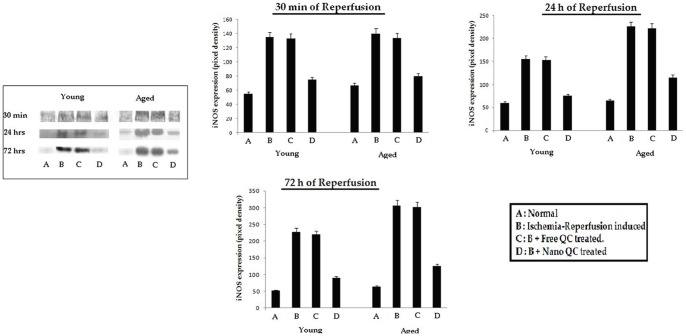
Western blot analysis to detect the effect of nanoencapsulated quercetin on iNOS release in the hippocampus of young and aged rats subjected to ischemia followed by reperfusion for 30 min, 24 h and 72 h. **A**. Normal, **B**. Ischemia-Reperfusion induced, **C**. **B**+ Free QC treated, **D**. **B**+ Nano QC treated. Values are mean ± SE of rats.

Inducible nitric oxide synthase (iNOS) is an enzyme that produces toxic levels of nitric oxide (NO) and is expressed in a number of brain pathologies, including cerebral ischemia. In our present study we observed that ischemic insults followed by respective intervals of reperfusion for 30 min, 24 h and 72 h resulted in the upregulation of iNOS protein expression in the hippocampus of experimental rats as observed from western blot analysis (Fig. 4). Free quercetin treatment could hardly check the increased expression of the protein whereas oral treatment with nanoencapsulated quercetin resulted in comparatively significant downregulation of iNOS expression in the hippocampus of both young and aged rats (Fig. 4).

### Effect of nanoencapsulated quercetin on Caspase-3 activity of ischemia-reperfusion induced hippocampus of young and aged rats

**Figure 5:**
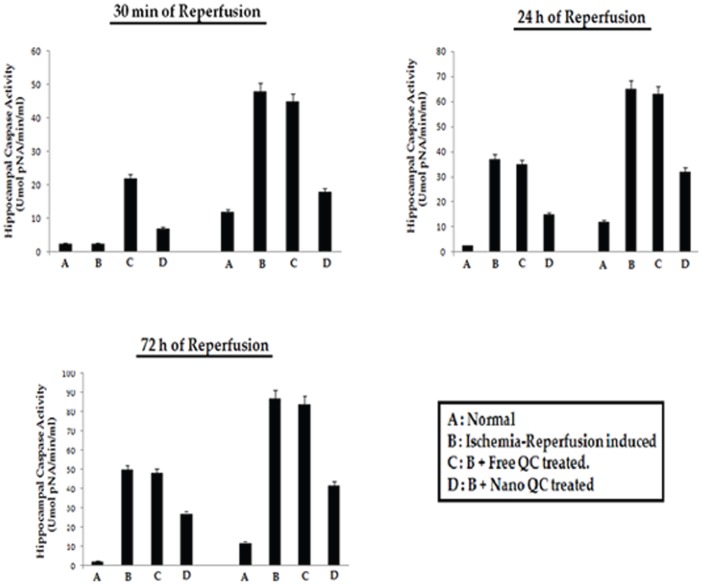
Effect of nanoencapsulated quercetin on caspase-3 activity in hippocampal region of ischemia-reperfusion induced young and aged rats. **A**. Normal, **B**. Ischemia-Reperfusion induced, **C**. **B**+ Free QC treated, **D**. **B**+ Nano QC treated. Values are mean ± SE of rats.

Caspase-3, an executioner caspase, is believed to play a vital role in apoptosis in a wide variety of cells. Activated caspase-3 cleaves proteins that are important in maintaining neuronal process integrity. Ischemic insult followed by reperfusion intervals of 30 min, 24 h and 72 h resulted in an increase in caspase-3 activity in the hippocampal region of both young and aged rats (Fig. 5). Free quercetin treatment resulted in no significant improvement whereas nanoencapsulated quercetin treatment prior to ischemic insult as well as post operation minimized the caspase-3 activity thus giving significant protection to the hippocampal region of both young and aged rats (Fig. 5).

### Effect of nanoencapsulated quercetin on pyramidal nerve cell count of ischemia-reperfusion induced hippocampal region of young and aged rats

**Figure 6:**
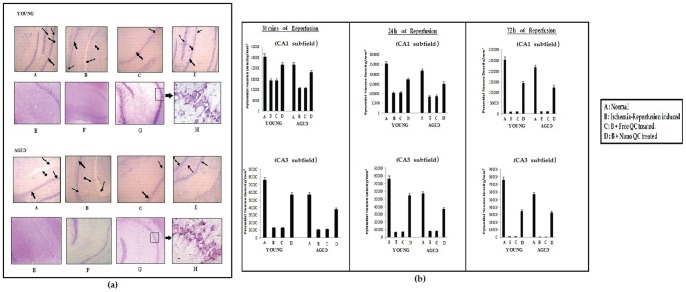
(a) Cresyl Violet stained sections of hippocampus of young and aged rats showing neuronal morphology in different hippocampal regions. A. Normal, B. Ischemia- Reperfusion (30 min) induced, C. B + Free QC treated, D. B+ Nano QC treated. E. Ischemia- Reperfusion (72 h) induced. F. E + Free QC treated; G. E + Nano QC treated., 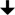, CA1 subfield; 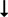, CA3 subfield; 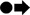, Dentate Gyrus; 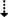, Hilus (b) Effect of NPQC treatment on neuron density of hippocampal regions (CA1 and CA3) of young and aged rats subjected to ischemia followed by reperfusion for 30 min, 24 h and 72 h. A. Normal, B. Ischemia- Reperfusion induced, C. B + Free QC treated, D. B+ Nano QC treated.

The hippocampal formation is the most frequently examined region in studies of the mechanism of ischemic cell death and selective neuronal vulnerability. A brief period of ischemic insults is known to cause cell death in hippocampal CA1 and CA3 pyramidal neurons. Analysis of the data from the control young and aged rat hippocampal brain region (Fig. 6) revealed that there was a significant effect of age on pyramidal neuron density in both CA1 and CA3 subfields of the hippocampus of the normal aged rats. Upon ischemic insult followed by reperfusion intervals of 30 min, 24 h and 72 h, there was a significant decline in the neuronal density in the CA1 and CA3 hippocampal subfields of both young and aged rats (Fig. 6b). The other two regions (Fig. 6a) viz. dentate gyrus and hilus also experienced nerve cell loss in both aged and young rat hippocampus. Free quercetin treatment hardly produced any improvement in the nerve cell count whereas nanoencapsulated quercetin treatment proved to be highly effective even post operation till day 3 in preventing hippocampal neuronal loss in both aged and young rats (Fig. 6).

## Discussion

Cellular damage arising from the oxidative stress (OS) has been implicated in neuronal degeneration associated with normal ageing [33], [34], [35]. Enhanced production of ROS and the subsequent oxidative stress have been thought to play a pivotal role in cerebral ischemia reperfusion. Amongst all the brain regions studied, the pyramidal neurons of the hippocampus is discerningly vulnerable to ROS mediated ischemic injuries and neuronal cell death [36]. Ischemic hippocampal pyramidal cell death both in rodents [37] and humans [38] is characterized by a selective neuronal loss that typically occurs over a period of 24–72 hours, a phenomenon known as delayed neuronal death. The present study addressed the efficacy of PLGA nanoparticles (NP) that might be authentically used to deliver polyphenolic bioflavonoid antioxidant, quercetin, to the brain across the blood brain barrier in a relevant rat model of ischemia followed by both short term (30 min) and prolonged (24h–72 h) reperfusion induced by common carotid artery occlusion.

The primary advantage of NP carrier technology is that NPs mask the BBB limiting characteristics of the therapeutic drug molecule. Furthermore, this system may also slow drug release in the brain, decreasing peripheral toxicity [39]. Poly (lactic-co-glycolic acid) (PLGA) is known to be one of the most successfully developed biodegradable polymers. It is biodegradable and biocompatible and it has the ability of controlling the release of drug that will extend blood circulation time. PLGA nanoparticles have been claimed to be excellent vehicles for delivery of a number of biomolecules, drugs and vaccines to the site of interest *in-vivo*. The size and size distribution of nanoparticles are important in determining their stability for drug release and cellular uptake efficiency [40]. Nanoparticles less than 100 nm in size have a higher potential to circulate in the blood for longer periods of time and experience reduced hepatic filtration. In the present study, the size range of the quercetin nanoparticles achieved as observed under the transmission electron microscope and atomic force microscope (Fig. 1) showed no aggregation, and all particles being below 100 nm makes the delivery system very much suitable for drug delivery to target organs, especially the brain, which has remained the ultimate challenge in drug delivery.

Lipid peroxidation is vulnerable to reactive oxygen radicals (ROS) induced by ischemia-reperfusion, which cause oxidative damage to brain bio membrane, lipids, proteins and DNA leading to brain dysfunctions and nerve cell death [41], [42]. Elevation of conjugated diene levels, the index of lipid peroxidation and cell damage, was reported in the rat cerebral cortex region after ischemia and reperfusion [43]. In the present study, table 1 showed that cerebral ischemia and subsequent reperfusion caused a subsequent increase in conjugated diene level in different brain regions of young and aged rats. Pre-treatment with nanoencapsulated quercetin significantly prevented increase in conjugated diene formation both in young and aged animals subjected to ischemic injuries (Table 1) whereas free quercetin treatment hardly showed any effect on the ischemia-reperfusion induced animals.

The antioxidant enzyme capacity of the brain tissue affected by ischemia-reperfusion is particularly important for the primary endogenous defence against the free radical induced injury [44] and involves the cooperative action of the intracellular antioxidant enzymes such as SOD and catalase. Reduced glutathione (GSH) participates in antioxidative defence by regenerating antioxidants [45] and by reducing hydroperoxide via the glutathione peroxidase cycle. In this study, determination of enzymatic antioxidant defence activity in young as well as aged rat brain regions was observed after cerebral ischemia and reperfusion. Nanoparticulated quercetin treatment prior to ischemic insult imparted absolute protection to enzymatic antioxidant systems (Fig. 2 and Table 2) whereas free quercetin hardly showed any level of improvement.

Brain cells require a constant supply of energy substrates to maintain ionic equilibrium across neural membranes [46]. Ischemia depletes brain cells of energy substrates. Cell membrane ionic pumps fail, leading to brain edema. Edema development resulted in the decrease in neuronal osmolality and loss of blood-brain-barrier integrity [47]. Cerebral ischemia resulted in a significant increase in brain cell water content (Fig. 3). Oral treatment with nanoencapsulated quercetin prior to ischemic insult controlled the osmolality in different brain regions of both young and aged rats (Fig. 3).

Reactive oxygen species have been implicated in the pathophysiology of many neurologic disorders and brain dysfunctions [48]. Recent studies have provided evidence that indirect signalling pathways by ROS can also cause cellular damage and death in cerebral ischemia and reperfusion. In the present study, rats subjected to cerebral ischemia-reperfusion showed increased ROS generation, hippocampus being the most affected brain region (Table 3). Nanoparticle encapsulated quercetin treatment prior to ischemic insult showed protective effects by minimizing ROS generation in both young and aged rat brain regions (Table 3). The decreased ROS must therefore be able to counterbalance the increased conjugated diene formation and depleted glutathione and antioxidant enzyme levels.

Several studies have shown that oxidative stress might lead to several alterations in membranes, including changes in the biophysical properties such as asymmetry and fluidity [49]. In the present study, the maintenance of mitochondrial membrane fluidity of the brain cells was observed to be achieved by the protective action of quercetin in nanocapsules in cases where a significant drop of mitochondrial membrane microviscosity occurred in different brain regions of young and aged rats due to the induction of ischemia-reperfusion (Table 4). Prior treatment with free quercetin showed no significant improvement in the membrane fluidity in different brain regions of ischemia-reperfusion induced rats.

Ischemic insults result in delayed neuronal death in selectively vulnerable brain regions such as the hippocampal subfields [50]. Distinct populations of hippocampal neurons are targeted by ischemia and multiple factors, including excitotoxicity, oxidative stress, and inflammation, and are responsible for their damage and death [51]. A number of recent studies showed that ischemia-reperfusion induced neuronal cell death involves apoptosis which is an active and genetically controlled cell suicide process [52]. Caspase 3 is known to be a potent effector of apoptosis. The participation of caspase-3 activation in the evolution of neuronal death after traumatic brain injury in rats was examined [53]. In the present study, caspase-3 activity appeared to be increased in selectively vulnerable brain region such as the hippocampal region of ischemia-reperfused young and aged rats (Fig. 5). Prolonged periods of reperfusion after 72 h resulted in remarkable increase in hippocampal caspase 3 activity (Fig. 5). Treatment with nanoencapsulated quercetin prior to ischemic insult as well as post-operation till day 3 checked the increased activity of caspase-3 in the hippocampal region of both young and aged rats (Fig. 5), thereby bringing alterations in the apoptotic processes that lead to neuronal cell death in the hippocampal region of the ischemia-reperfused animals.

Excessive release of nitric oxide (NO) has been implicated in pathophysiology of neurodegeneration in ischemic stroke [54]. Therefore, inhibition of iNOS might pave a new way in understanding the novel therapeutic strategy targeted specifically at the secondary progression of ischemic brain injury. In the present study western blotting showed the overall expression of iNOS in the hippocampal region of ischemia-reperfusion induced young and aged rats tend to increase with the increased duration of reperfusion. The expression of iNOS reached its highest peak after 72 h of reperfusion (Fig. 4). Maximum protection against ischemia-reperfusion induced iNOS expression in the hippocampal brain region was observed in those groups of rats that were orally treated with nanoencapsulated quercetin (Fig. 4) post-operation and also prior to ischemic insults (Fig. 4).

Upregulation of iNOS expression leads to excessive nitric oxide (NO) production. This excess NO reacts with superoxide to form peroxynitrite, a powerful radical that induces neuronal death after cerebral ischemia [50]. In the present study a significant reduction in the pyramidal neuronal density in the CA1 and CA3 hippocampal subfields of ischemia-reperfusion induced young and aged rats (Fig. 6) was observed. The severity in both the ischemic hippocampal subfields was observed to be maximum when the reperfusion time was extended till 72 h (Fig. 6). However, the hippocampal neuronal damage was to some extent prevented upon oral treatment with nanoencapsulated querctin, 2 h prior to ischemic insult in both young and aged rats (Fig. 6). The diminution of hippocampal pyramidal neuronal counts was further checked upon extending the oral treatment of nanoencapsulated quercetin post operation for 3 more days (Fig. 6). A majority of the post-operative death was reported to occur after 3 days of ischemia. No animals died after 30 min of ischemia followed by 30 mins of reperfusion. 50% mortality was reported after 24 h of reperfusion especially with the aged population of rats. However, nanoencapsulated querectin treated animals, irrespective of young and aged groups, survived till day 3 of reperfusion.

## Conclusion

Based on the results presented herein, we propose that oral treatment with nanoencapsulated quercetin might play a protective role against oxidative damage by preventing the loss of pyramidal neurons from the hippocampal CA1 and CA3 subfields in ischemia reperfusion induced young and aged rats. However, shorter durations of reperfusion produce significantly less damage than longer durations and early treatment with nanodrugs increase the chances of survival owing to reduced neuronal damage. This approach of delivering a non-toxic herb origin antioxidant, quercetin, to brain offers the potential clinical application in human neurodegenerative diseases in future.
